# Sleep and health-related physical fitness in children and adolescents: a systematic review

**DOI:** 10.5935/1984-0063.20200125

**Published:** 2021

**Authors:** Ana Paula Leão Maia Fonseca, Carolina Virginia Macêdo de Azevedo, Rute Marina Roberto Santos

**Affiliations:** 1 Universidade Lusófona de Humanidades e Tecnologias, Faculdade de Educação Física e Desporto - Lisboa - Portugal.; 2 Universidade Federal do Rio Grande do Norte, Departamento de Fisiologia e Comportamento - Natal - Rio Grande do Norte - Brazil.; 3 University of Porto, Research Centre in Physical Activity, Health and Leisure, Faculty of Sport - Porto - Portugal.; 4 General Directorate of Health, National Program for Physical Activity Promotion - Lisbon - Portugal.

**Keywords:** Sleep, Adolescents, Children, Physical fitness, Sleep duration, Sleep quality

## Abstract

**Objectives:**

The aim of this systematic review was to summarize the evidence on the associations between the sleep duration or sleep quality and cardiorespiratory and muscular fitness in children and adolescents aged 6-19 years.

**Material and Methods:**

This systematic review followed the Preferred Reporting Items for Systematic Reviews and Meta- analyses (PRISMA) and was registered with the international prospective register of systematic reviews PROSPERO network. Three databases (PubMed, SPORTDiscus and Science Direct) were searched until October 2019 for scientific articles concerning sleep duration, sleep quality and physical fitness.

**Results:**

Six articles, including 5797 participants, from 11 different countries, were included in the current systematic review.

**Conclusion:**

Longer periods of sleep and better sleep quality were associated with higher levels of physical fitness.

## INTRODUCTION

Physical fitness is the capacity needed by an individual to execute specific motor abilities with vigor and alertness, without undue fatigue, and with ample energy to enjoy leisure-time pursuits and meet unforeseen emergencies. Physical fitness capacities are usually grouped into health-related (cardiorespiratory fitness, body composition, muscular strength, and endurance and fexibility) and skill-related components (agility, coordination, balance, power, reaction time, and speed)^1^. High levels of cardiorespiratory and muscular fitness have been associated with lower cardiovascular disease risk, better quality of life, and positive health in youth^2, 3^. However, adolescents’ physical fitness levels have declined over the last decades^4^.

Sleep is a basic human need, with important physical and mental health and well-being implications^5, 6^, and chronic sleep loss is an important public health issue^7^. Inappropriate sleep duration has been associated with several health implications. In children and adolescents, evidence suggests that short sleep duration is associated with excess adiposity, poor emotional regulation (stress, anxiety, and depressive symptoms), lower academic achievement (e.g., concentration and memory) and lower quality of life/well-being^8^.

The National Sleep Foundation in the USA recommends that children and adolescents aged 6-13 years should sleep between 9-11h per night, and adolescents aged 14-17 years need 8-10h per night to maximize general health^9^. However, despite the detrimental health effects of inappropriate sleep, evidence shows that children and adolescents tend to sleep less compared with previous generations^10, 11^.

Adolescence (aged 10 to 19 years) is a stage of life characterized by many behavioural and physiological changes^12^. Adolescence begins with the onset of puberty, a phase of life in which there are changes to the physical appearance and reproductive capacity, which impact adolescents’ psychological and social development. This is also a period of new rights and responsibilities, development of autonomy and identity, as well as relational changes with family, friends, and school^13^.

During adolescence there is a higher sleep necessity than in adulthood, but lower than during childhood^14, 15, 16^. However, it is common to observe short sleep duration among adolescents due to a conflict between the biological delay of sleep times^14, 15, 17^ and social factors^18^, such as morning school schedules, which act as a strong synchronizer of wake-up time over the week^14^. Thus, adolescents wake up earlier, shortening their sleep duration during the week, and wake up later, extending their sleep on weekends. This leads to irregular sleep times and durations^19^. This disruption might be exacerbated by other social factors that delay sleep onset, such as: (i) more autonomy regarding the sleep times^20^, interaction with social networks^21^; (ii) an earlier wake time to comply with academic activities^17^; (iii) increased use of electronic media^22, 23^ and increased light exposure at night related to these activities^22, 24, 25^. During adolescence short sleep duration may also be related to pubertal stage, as more mature adolescents show later sleep times^17^. It is hypothesized that late sleep times result from modifications to the SWC (sleep-wake cycle) regulation processes^7, 26^, which affect the expression of the circadian rhythm in adolescents^19^.

Sleep duration has been associated with physical fitness levels. For those who are sleep deprived, increasing sleep has shown to improve multiple dimensions of function^27^. In athletes, it has been reported that sleep restoration and sleep extension improves tennis-serve accuracy^30^, running and swim sprint times^28, 29^, swim-turn and kick-stroke efficiency^28^, basketball shooting accuracy, half-court and full-court sprints^31^, and time to exhaustion^32^. Most studies agree on recommending athletes to increase their sleep by 2h (with a goal of up to 9h for elite athletes)^28, 29, 30, 31, 33^, as sleep is an unquestionably vital physiological function and one of the most important factors in exercise recovery^34^.

A recent study has shown that lower levels of physical fitness and ‘insufficient’ physical activity are associated with poor sleep quality in a large sample of young adults^35^. In adolescents from 11-16 years old, the engagement in moderate-to-vigorous physical activity has been associated with better sleep quality, but compared to boys, girls had lower levels of physical activity and poor sleep quality^36^. Some studies have reported the beneficial effects of physical fitness and physical activity in promoting falling sleep and ‘good’ sleep quality^37^. Likewise, poor sleep quality has been associated with lower levels of muscular endurance, flexibility, and cardiorespiratory fitness in young adults^38, 39^.

In this context, the aim of this systematic review is to summarize the knowledge on the association between sleep duration or sleep quality and health-related physical fitness (cardiorespiratory and muscular fitness) in children and adolescents.

## MATERIAL AND METHODS

### Protocol and registration

This systematic review followed the preferred reporting items for systematic reviews and meta-analyses (PRISMA)^40^ and was registered with the international prospective register of systematic reviews PROSPERO (CRD42020199083).

### Study selection criteria

Three electronic databases were searched from the inception until October 2019 (PubMed, SPORTDiscus and ScienceDirect).

For the present systematic review, we considered only observational studies (cross-sectional and longitudinal) as well as intervention studies reporting results from baseline data (age criteria applied) were considered; methods/protocol papers, conference papers, editorials, commentaries, and reviews were not considered.

Studies with apparently healthy children and adolescents aged 6-19 years old (including overweight or obese) that had been published in English, Spanish or Portuguese were considered. We also checked the reference lists of the papers included, as well as relevant reviews to identify other potential studies. We checked the study against the priori determined PICO (population, intervention, comparator, and outcome) study criteria^41^. Studies reporting interventions with sleep hygiene and sleep patterns (variables as sleep duration or bedtimes and wake-up times and sleep quality), physical fitness and the association between sleep duration and physical fitness, were included.

### Data extraction

Selected studies were imported to the manager software (Mendeley 1.18), and duplicated were removed. One author (APF) screened titles and abstracts independently and disagreements was decided by discussion and consulting the second and third authors CA and RS.

### Data sources and search strategy

Three electronic databases were searched from the inception until October 2019 (PubMed, SPORTDiscus and ScienceDirect). The search strategy for PubMed was (((“Cardiorespiratory Fitness” [Mesh] OR “Cardiovascular Fitness” [Title/Abstract] OR endurance [Title/Abstract] OR “aerobic fitness” [Title/Abstract] OR “aerobic endurance” [Title/Abstract] OR “Muscle Strength” [Mesh] OR “Physical Conditioning, Human” [Mesh] OR “Endurance Training” [Mesh]))) AND (((sleep [Title/Abstract]) OR “sleep duration” [Title/Abstract])). This search strategy was then adapted to SPORTDiscus and ScienceDirect.

### Risk of bias assessment

Risk of bias criteria was adapted from STROBE (STrengthening the Reporting of Observational studies in Epidemiology)^42^, which has been previously applied in systematic reviews^41, 43, 44, 45^. The criteria for risk bias were the following: (1) Was eligibility criteria applied?; (2) Were participants selected randomly?; (3) Did the participants represent a specific population? (that is, country or region)?; (4) Is the sample size of the study larger than 100?; (5) Were the source and details of the variables used to evaluate sleep and physical fitness reported? (6) Were the measurements used valid and reliable to adolescents or children? An amount of 1 (yes) or 0 (no or unsure) has been assigned to the answer of every of these questions allowing for a maximum possible score to 6 points. Studies with scores from 3 to 5 were classified as having a lower risk of bias (Table 2).

### Data analysis and synthesis

Given the heterogeneity of the included studies, a meta-analysis was not possible. Therefore, we conducted a narrative summary of the results and the characteristics of each study

## RESULTS

**Figure 1:**
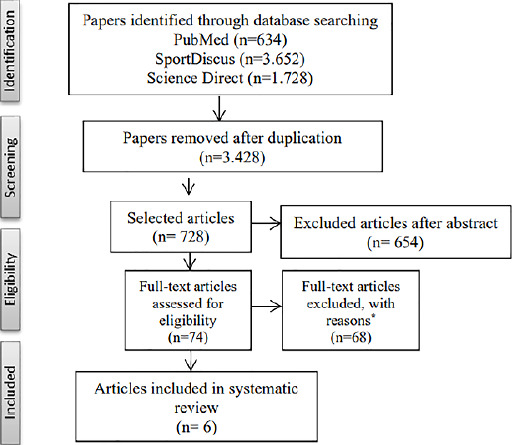
Flow chart of studies selection process. * Reasons described in the exclusion criteria.

The systematic search identified 6,014 potential articles. After exclusions based on duplication of articles, 728 remained for abstract screening. Then, 654 articles were excluded, of these 74 full texts were screened and 68 were excluded for reasons such as the subjects’ age, variables of interest or lack of association between sleep and physical fitness. Thus, a total of 6 studies were included in this review (Figure 1).

The number of participants within the included studies was 5,797, and the studies had the following designs: cross-sectional (n=4), follow-up (n=1) and cohort (n=1). All studies were published between 2007 and 2016, and the sample sizes ranged from 236 to 4,903 subjects. The number of studies by country was: United States (1), France (1), Taiwan (1), Portugal (1), Chile (1), and one was conducted with a multicountry sample of participants from Belgium, Cyprus, Estonia, Germany, Hungary, Italy, Spain, and Sweden. The characteristics and results of each of the included studies are presented in Table 1.

**Table 1:** Summary of included studies.

Author & date	Study Design	Sample size	Girls	Age	Location	Tools	Outcome measures	Absolute effect
Lee & Lin, 2007^38^	Cross-sectional	291	291	19.3 ± 0.6 yrs	Taiwan	Digital Height-Weight measurement; Pittsburgh Sleep Quality Index (PSQI) Questionnaire; Sit and reach test, curl-up test and 800m run/walk test;	BMI; Sleep quality evaluation (sleep quality, sleep onset latency, sleep duration, sleep efficiency, sleep disturbances); Physical fitness (cardiovascular endurance, body composition, muscular strength and endurance, fexibility)	Sleep duration was negatively correlated with 800m run/walk test (r=-0.34; p<0.05) and with the sit-andreach test (r=-0.24; p<0.05). Thus, there is a significant relationship between sleep duration and cardiovascular fitness and flexibility. Subjects with poor sleep quality (PSQI score >5) showed less sleep duration (t-test=9.57; p<0.05) and were more likely to have lower levels of muscular endurance (t-test =4.42; p<0.05), flexibility (t-test=-5.12; p<0.05), and cardiovascular fitness (t-test=7.27; p<0.05) than subjects with good sleep quality.
Countryman et al, 2013^46^	Cohort (2000-2005)	367	99	16.1 ± 0.7 yrs	EUA	Mercury sphygmomanometer; Balance beam scale and height rod; Waist circumference; Blood collection; Self-report of sleep duration, quality and fatigue; Maximal treadmill test (modified Balke (walk–jog) exercise protocol);	BMI, Blood pressure, Fasting Blood Measures (serum cholesterol, triglycerides, lipoproteins, glucose, insulin, fibrinogen, high-sensitivity CRP and IL-6); Sleep duration; Children’s Depression Inventory; Seven-Day Physical Activity Recall; Aerobic fitness (peak VO2);	The sleep may in part influence cardiometabolic outcomes through associations with fitness. Specifically, reduced sleep duration (7.7h ±1.2) and a composite score based on poor sleep quality and fatigue were associated with decreased cardiorespiratory fitness, which was in turn related to increased risk of metabolic syndrome and inflammation. The sleep latent variable was positively associated with aerobic fitness (coefficient=2.52, z=2.67, p=0.01).
Thivel et al, 2015^47^	Cross-sectional	236	224	7.5 ± 0.6 yrs	France	Anthropometric parameters (stadiometer, Digital scale Seca model 873, waist circumference); BMI; Skinfold thickness (skinfold caliper); Children’s Eating Habits questionnaire; Self-report (parental factors); Cardiorespiratory fitness (20-m shuttle run test (20-MST); musculoskeletal (squat jump and cycling peak power) fitness (cycle ergometer; Plateform Ergo Jump PlusF Bosco System).	Anthropometric Characteristics (Body weight, Height); Body composition (BMI, Index of adiposity); Eating habits; Sleep patterns (bedtime, wake-up time and sleep duration); Physical Fitness (cardiorespiratory and, Musculoskeletal Fitness, Squat jump - lower limb explosive strength);	Neither cardiorespiratory fitness level nor musculoskeletal fitness level were significantly different between late and normal sleepers and none of the physical fitness parameters was associated with sleep duration, bedtime and wake-up time (p>0.05 for all).
Zaqout et al, 2016^48^	Follow up (2006-2012)	4903	2481	8.7 ± 1.2 yr	Belgium, Cyprus, Estonia, Germany, Hungary, Italy, Spain, Sweden	TANITA scale and a portable stadiometer; Questionnaire from parental-reported bedtime and get-up time of children, separately for weekdays and weekends, self-report (parental factors); Proxy-reported KINDL for parents, Food Frequency Questionnaire. ALPHA health-related fitness test battery, Actigraph accelerometers Anthropometric measurement;	Sleep duration and Parental factors; Psychosocial well-being, Dietary habits. Physical activity, Physical fitness (cardio-respiratory, muscular strength, flexibility, balance and speed)	There was no significant association between sleep duration and cardiorespiratory fitness, muscle strength, speed, flexibility and balance levels (p> 0.05 for all).
Mota & Vale, 2010^50^	Cross-sectional	886	886	15.4 ± 1.9 yrs	Portugal	Electronic weight scale (portable digital beam scale; stadiometer); Quality of Sleeping Time (QST) status by responding to the question, ‘‘In general, how is your sleeping time?’’ Items are scored on a Likert scale with 1 ‘‘poor’’ to 5 ‘‘excellent.’’ Maximal multistage 20 m shuttle-run test according to procedures described from FITNESSGRAM; Shuttle Run Test.	Anthropometric measures (height, weight); BMI; Quality of Sleeping Time (QST) CRF (cardiorespiratory fitness – aerobic capacity)	Statistically significant association was observed between the QST and CRF (Rho=0.17; p < 0.05). Poor sleep quality in adolescent girls was associated with lower CRF. Additionally, girls who were classified as fit were twice as much higher odd to report better sleep quality compared to their unfit peers.
García-Hermoso et al, 2015^49^	Cross-sectional	395	196	12.1 ± 0.7 yrs	Chile	Digital scale and stadiometer; Self-report (Sleep self-report Spanish version; self-reported screen time, self-reported physical activity -Questionnaire for Adolescents (PAQ-A) and socioeconomic status); 20-m shuttle-run test (Alpha Battery ).	BMI (Weight, Height, waist circumference); Sleep patterns (sleep quality, sleep-related anxiety, bedtime refusal, and sleep routines) CRF (cardiorespiratory fitness); PA (physical activity); Screen Time	In both sexes, sleep-related anxiety problems were correlated with CRF (boys, r=-0.202, p<0.05; girls, r=- 0.0064, p<0.05). However, sleep quality was correlated with CRF only in girls (r=-0.0065, p<0.05). A higher CRF level was associated with a lower likelihood of having sleep-related anxiety problem in girls (OR = 0.20, 95% CI, 0.01 to 0.53, p = 0.031).

All studies were classified as having a low risk of bias. Table 2 shows the scores of each study for risk of bias.

**Table 2:** Risk of Bias of the included studies.

	Q1 (Was eligibility criteria applied?)	Q2 (Were participants selected randomly?)	Q3(Did the participants represent a specific population? (that is, country or region)	Q4 (Is the sample size of the study larger than 100?)	Q5 Were the source and details of the variables used to evaluate sleep and physical ftness reported?	Q6 (Were the measurements used valid and reliable to adolescents or children?)	Score final
Lee and Lin (2007)^38^	1	0	0	1	1	1	4
Countryman et al. (2013)^46^	1	0	1	1	1	1	5
Thivel et al. (2015)^47^	1	0	1	1	1	1	5
Zaqout et al. (2016)^48^	1	0	1	1	1	1	5
Mota and Vale (2010)^50^	0	0	1	1	1	1	4
García-Hermoso et al. (2015)^49^	1	0	1	1	1	1	5

Notes: Q = Question; Yes = 1; No or doubt = 0.

Sleep duration showed a significant relationship with cardiorespiratory fitness and fexibility. Reduced sleep duration in adolescents was associated with decreased cardiorespiratory fitness, lower levels of muscular endurance, and fexibility when compared to adolescents with appropriate sleep duration^38, 46^. However, in children, no significant differences were found for cardiorespiratory and musculoskeletal fitness levels between late and normal sleepers. Further, none of the physical fitness parameters was associated with sleep duration, bedtime or wake-up time^47^. In addition, there was no significant association between sleep duration and cardiorespiratory fitness and muscle strength, nor between speed, agility, and flexibility^48^.

Higher cardiorespiratory fitness was associated with a lower likelihood of having a sleep-related anxiety problem in adolescents^49^. Additionally, girls who were classified as fit were twice as likely to report better sleep quality than their unfit counterparts. However, girls with poor sleep quality showed lower cardiorespiratory fitness^50^. Moreover, poor sleep quality and short sleep duration in girls were associated with lower levels of muscular endurance, flexibility, and cardiovascular fitness^38^.

## DISCUSSION

The aim of this systematic review was to summarize the knowledge on the association between sleep duration or quality and physical fitness (cardiorespiratory and muscular fitness) in children and adolescents. The National Sleep Foundation in the USA recommends that children aged 6-13 years should sleep between 9-11h per night and that adolescents aged 14-17 years sleep 8-10h per night to maximize general health^9^. However, it is often observed that short sleep duration among adolescents results from a conflict between the biological delay on sleep times^14, 15, 17^ and such social factors^18^ as morning school schedules, which act as a strong wake-up time synchronizer across the week^14^.

Our results suggest that reduced sleep duration was associated with decreased cardiorespiratory fitness, lower levels of muscular endurance and flexibility in adolescents^38, 46^; however, no associations were found between sleep duration and cardiorespiratory fitness or muscle strength among a large sample of European children aged 6-9 years old^47, 48^. While these studies reported null findings^47, 48^, more research exploring this relationship in children in other countries and regions and with wider age ranges are required before a clear conclusion can be reached.

In addition, it is important to notice that ideal sleep entails not only an adequate amount of sleep, but also other sleep features such as sleep architecture (i.e., sleep stages), sleep quality (i.e., efficiency to stay asleep), time (i.e., time to sleep/wake up), consistency (i.e., day by day variability), and continuity (that is, variability in sleep duration within the same night)^5^. For example, the results of this systematic review point to an association between poor sleep quality and lower cardiorespiratory fitness in adolescent girls^50^. Moreover, girls with poor sleep quality and shorter sleep duration were more likely to show lower muscular endurance, flexibility, and cardiovascular fitness^38^. Conversely, a higher cardiovascular fitness was associated with a lower likelihood of having sleep-related anxiety problems in adolescents^49^. Additionally, girls who were classified as fit had twice the odds of reporting better sleep quality compared to their unfit counterparts^50^.

The results of the present systematic review are in line with previous studies showing a positive association between physical activity or fitness and sleep parameters (subjective sleep/sleep disturbance, sleep quality, shortened sleep onset latency, sleep onset, and total sleep time)^36, 50, 51^ in adolescents. However, the association between physical fitness and sleep duration or sleep quality in adolescents has not been extensively studied. One important aspect to be taken into account about the effects of sleep duration on the athletic performance of adolescents is the occurrence of naps. The studies that were evaluated in this review only considered nocturnal sleep. A study has shown that shorter nocturnal sleep duration may be influenced by a high frequency of daytime napping^52^. This may be due to the fact that adolescents with shorter nighttime sleep duration may try to catch up on sleep during the day. So, does the teenager who sleeps less at night and naps during the day attenuate the negative effects on cardiorespiratory fitness because the nap compensates for some of the sleep deprivation?

Another aspect is that physical activity has been considered an effective, non- pharmacological approach to improve sleep. However, physical activity and sleep assessment methodologies still present challenges. Additionally, the comparisons of results between studies is often problematic due to the multiplicity of assessment tools, data analyses and reporting procedures^53, 54^. Adolescence is a period of life when multiple changes occur in the physiological, psychological, psychiatric, and socio cultural domains^55^. When constructing a self-reported assessment tool for this age group, the option to place emphasis on either quantitative or qualitative aspects of physical activity and fitness and sleep is paramount.

In this context, increased physical inactivity among children and adolescents^56, 57, 58^ is worrisome. In the past few years, there has been a steep increase in research on the health-related effects of physical inactivity and sedentary behavior^59^.

Notably, a recent systematic review reported consistent evidence for a positive association between muscular fitness and physical activity^60^; however, given the limited number of available studies, the association between muscular fitness and sleep was considered uncertain. This is also in line with a previous systematic review by Poitras et al. (2016)^61^, with evidence suggesting that moderate-intensity physical activity may be sufficient for improving or maintaining cardiorespiratory fitness in children and adolescents. These findings continue to support the importance of encouraging physical activity during childhood and adolescence, according to the recommendations of the Physical Activity Guidelines Advisory Committee scientific report^62^, to mitigate against the consequences of the physical inactivity, sleep deprivation and poor sleep quality.

A few studies have shown that exercise improved sleep quality or duration, and sleep and exercise exert substantial positive effects on one another^58^. Importantly, previous research has reported the independent and additive effects of muscular and cardiorespiratory fitness on children and adolescents’ health^2, 63^, advocating that these fitness components may influence health through unique physiological pathways. However, the mechanisms involved in the relationship between physical fitness and sleep quality and duration are unknown. Thus, additional studies are necessary to understand these mechanisms.

Studies have also shown that there is a need for action. The lack of sleep and difficulty in sleeping well is a widespread health concern^58^ compounded by the fact that physical inactivity has increased among children and adolescents^56, 57, 58^. Adolescents are not getting as much sleep as recommended. In the last few years, we have observed a discussion to push for policy change in order to delay the time at which schools start in the morning^14, 64, 65^, as many adolescents wake up in the morning to go to school without having an adequate amount of sleep in the previous night, which can affect their school performance^8^, physical fitness, health and well-being.

Results showed that longer nocturnal sleep periods and better sleep quality were associated with higher levels of physical fitness in adolescents. However, such association was not observed in children. Further research is needed, for example through long-term observational and intervention studies that evaluate cause-and-effect relationships and use objective measures of sleep and physical fitness with greater representativeness among children and adolescents, exploring aspects of human lifestyle, differences between genders, age groups and cultural contexts.

The present review provides new contributions to this field of literature and supports the view that sleep duration and sleep quality are meaningfully linked with cardiorespiratory fitness and/or muscular fitness in adolescents.

### Strengths and limitations

To the authors’ knowledge, the present systematic review is the first to report on association between sleep duration, sleep quality, and physical fitness in children and adolescents. However, the scarcity of studies evaluating the physiological reasons for the effects of physical fitness upon sleep might occur was a limitation in our results. To better inform the association between sleep quality, sleep duration and physical fitness there is a need for further studies comparing objectively measured sleep outcomes and self-report ones.
